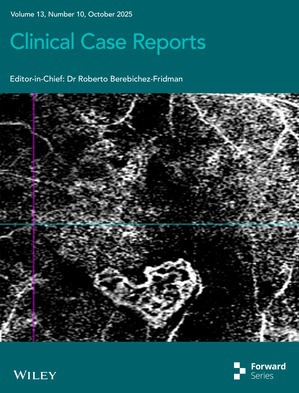# Cover Image

**DOI:** 10.1002/ccr3.71379

**Published:** 2025-10-23

**Authors:** Shangkun Ou, Feiping Shi, Minqing Cai, Yiming Wu

## Abstract

The cover image is based on the article *Conbercept Treatment for Heart‐Shaped Vascular Intertwined Nets in Macular Neovascularization: Anti‐VEGF Drug Therapy Strategy Based on Vascular Geometry Diagnosed by OCTA* by Shangkun Ou et al., https://doi.org/10.1002/ccr3.71250.